# Spatial variation of the gut microbiome in response to long-term metformin treatment in high-fat diet-induced type 2 diabetes mouse model of both sexes

**DOI:** 10.1080/19490976.2023.2188663

**Published:** 2023-03-16

**Authors:** Laila Silamiķele, Rihards Saksis, Ivars Silamiķelis, Patrīcija Paulīne Kotoviča, Monta Brīvība, Ineta Kalniņa, Zane Kalniņa, Dāvids Fridmanis, Jānis Kloviņš

**Affiliations:** Human genetics and disease mechanisms, Latvian Biomedical Research and Study Centre, Riga, Latvia

**Keywords:** Metformin, gut microbiome, intestinal parts, lumen and mucosa, C57BL/6N, high-fat diet, 16S rRNA sequencing

## Abstract

Antidiabetic drug metformin alters the gut microbiome composition in the context of type 2 diabetes and other diseases; however, its effects have been mainly studied using fecal samples, which offer limited information about the intestinal site-specific effects of this drug. Our study aimed to characterize the spatial variation of the gut microbiome in response to metformin treatment by using a high-fat diet-induced type 2 diabetes mouse model of both sexes. Four intestinal parts, each at the luminal and mucosal layer level, were analyzed in this study by performing 16S rRNA sequencing covering six variable regions (V1-V6) of the gene and thus allowing to obtain in-depth information about the microbiome composition. We identified significant differences in gut microbiome diversity in each of the intestinal parts regarding the alpha and beta diversities. Metformin treatment altered the abundance of different genera in all studied intestinal sites, with the most pronounced effect in the small intestine, where *Lactococcus* increased remarkably. The abundance of *Lactobacillus* was substantially lower in male mice compared to female mice in all locations, in addition to an enrichment of opportunistic pathogens. Diet type and intestinal layer had significant effects on microbiome composition at each of the sites studied. We observed a different effect of metformin treatment on the analyzed subsets, indicating the multiple dimensions of metformin’s effect on the gut microbiome.

## Introduction

Despite being widely studied, metformin is an antidiabetic drug with a still debated molecular action mechanism. Many studies have shown that its action is at least partially effectuated via the gut microbiome.^1–3^⁠ Gut microbiome composition and density vary along the gastrointestinal tract longitudinally and cross-sectionally.⁠^4^ Components contributing to this diversity include nutrient availability, chemical gradients, oxygen levels, mucosal structure, the diameter of the lumen, and cellular composition.^5^⁠⁠⁠

The gut microbiome is dominated by four phyla – *Bacteroidetes* (Gram-negative, anaerobic), *Firmicutes* (Gram-positive, anaerobic or obligate or facultatively aerobic), *Actinobacteria* (Gram-positive, anaerobic), and *Proteobacteria* (Gram-negative, aerobic or facultative anaerobic) in both mice and humans.^6,7^ The functional niche for each of these phyla is different as *Bacteroidetes* mainly produces acetate and propionate, but *Firmicutes* – butyrate.⁠⁠^8^

Functional differences between distinct regions of the gut correspond to the distribution of the microbial genera with respect to both identity and abundance. Studies in mice, piglets, and humans have shown that the proximal part of the small intestine – duodenum-jejunum, which is characterized by faster transit and facilitation of simple sugar and amino acid metabolism, is dominated by facultative anaerobes *Lactobacillus*, *Proteobacteria*, and obligate anaerobes *Bacteroides*, distal small intestine – ileum by *Fusobacterium* and *Escherichia* and the cecum and colon where the passage is slower and metabolism advantages fermentation of complex polysaccharides arising from undigested fibers or host mucus is prevailed by saccharolytic *Bacteroidales*, *Clostridiales*, and *Prevotella*.^4,9,10^⁠⁠⁠⁠

In healthy mice, *Lactobacillaceae* dominates the stomach and small intestine, while in the large intestine, the most abundant families are *Bacteroidaceae*, *Prevotellaceae*, *Rikenellaceae*, *Lachnospiraceae*, and *Ruminococcaceae*.^11^⁠ In the cecum of high-fat diet (HFD)-fed mice, the higher abundance of *Lachnospiraceae*, *Blautia*, and *Oscillibacter*, among others, has been shown; in addition, the colon and feces of HFD-fed mice are enriched in *Bacteroides* and *Proteobacteria* compared to control diet (CD)-fed ones.^12,13^⁠

Microbial communities differ on the transverse axis as well. Bacteria associated with the gut mucosa remain located at the same place; thus, these depend on the available substrate, whereas bacteria in the gut lumen can freely associate with various substrates. The mucosa is inhabited by aerotolerant taxa such as *Proteobacteria* and *Actinobacteria*, *Clostridium*, *Lactobacillus*, *Enterococcus*, and *Akkermansia*, while other bacteria predominate toward the lumen – *Bacteroides*, *Bifidobacterium*, *Streptococcus*, *Enterobacteriaceae*, and *Ruminoccoccus* among others.^4,14^⁠⁠ The mucosa-associated microbiota varies in different intestinal parts. Although fecal samples representing luminal content can provide information on global microbiota composition to a certain extent, research on mucosa-associated microbial communities can provide critical information on the interaction between gut microbiota and host.^15^⁠⁠

Absorption of metformin has been shown to vary between different intestinal sites, with the most prominent absorption occurring in the proximal small intestine.^16^⁠ Half of the administered dose reaches the distal small intestine, accumulating in the mucosal layer, and 30% of the dose is eliminated in the feces.^17^⁠ Due to various absorption rates in different parts of the gastrointestinal tract, it can be expected that the metformin’s effect on microbiome composition and the abundance of different microorganisms is not uniform. Furthermore, metformin accumulates in mucosal tissue,^18^⁠ thus the exposition time and presumably the effect on resident bacteria differ in various sites. These differences necessitate an in-depth analysis of the effect of metformin treatment on the gut microbiome composition at various gastrointestinal locations and layers.

The effect of metformin treatment has mainly been studied in cecal and fecal samples only, providing only limited information about the various aspects of the interaction between the gut microbiome and metformin. Short-term effects of metformin have been shown in different parts of the small intestine in the luminal layer in a recent study.^19^⁠ Our study adds information about the long-term effects of metformin therapy on different intestinal segments, not only in the luminal layer but also in the gut mucosa. Substantial advantages of our research include the analysis of both sexes and sequencing of six variable regions of the 16S rRNA gene, providing novel and trustworthy information on metformin’s effects. In addition, we employed a factorial animal experiment, including all the relevant control groups, allowing us to analyze the effect of various factors that potentially influence the interaction between metformin and the gut microbiome. Knowledge of the spatial variation of the gut microbiome in response to metformin treatment could potentially allow for more targeted microbiome modifications. In the future, this could alleviate the side effects experienced by metformin users, thereby increasing the applicability of the medication.

## Results

### Microbial composition analysis

**Figure 1.**
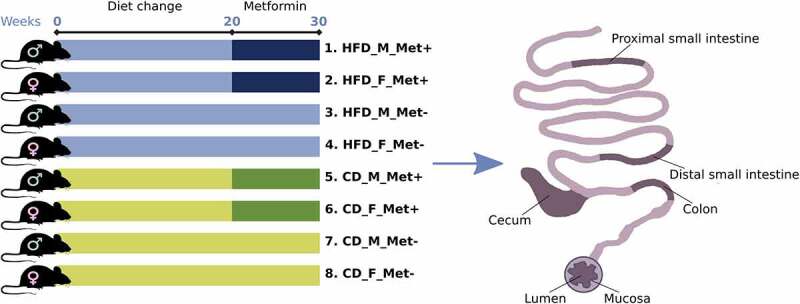
Experimental design of the study (*N* = 24) and intestinal sites studied. Abbreviations: HFD – high-fat diet; CD – control diet; M – male; F – female; Met+ – receiving metformin treatment for 10 weeks; Met- – not receiving metformin treatment.

In total, 192 microbiome samples representing four different intestinal regions at the luminal and mucosal layers collected from 24 mice were sequenced. One of the samples was excluded from further analysis due to possible mislabeling. Mice representing three experimental units were included in each group representing each of the eight treatment arms described in this study (Figure 1).

**Figure 2.**
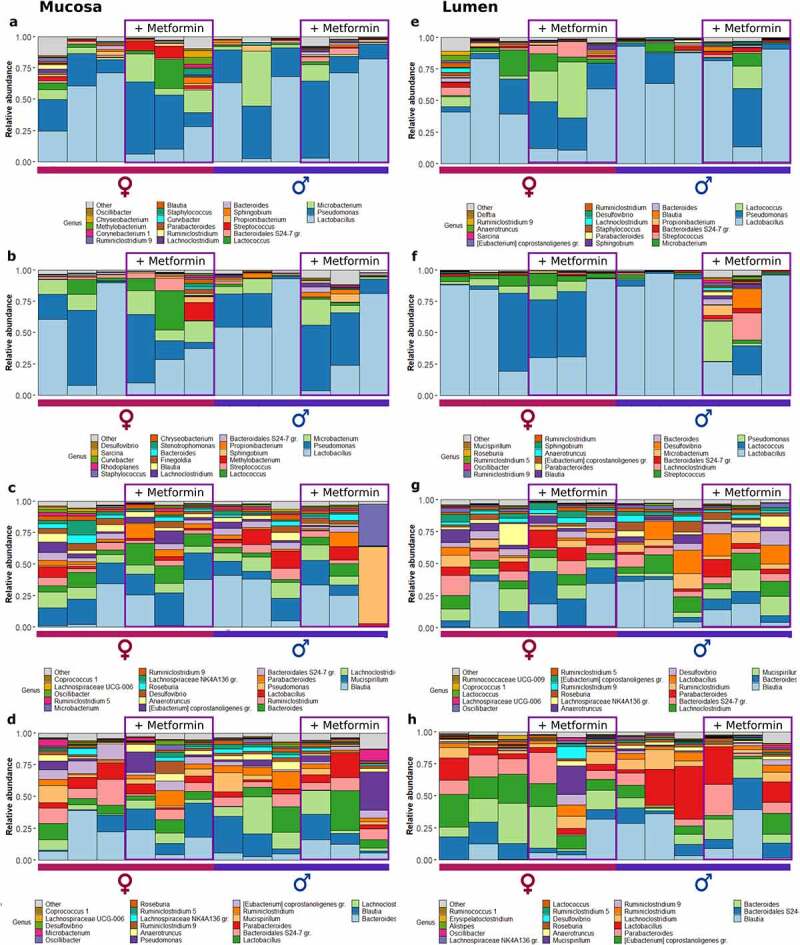
Microbiome composition at different sites of high-fat diet-fed mice at the genus level – top 20 genera are shown. Mucosal layer: (a) proximal small intestine; (b) distal small intestine; (c) cecum; (d) colon. Luminal content layer: (e) proximal small intestine; (f) distal small intestine; (g) cecum; (h) colon. Samples representing mice receiving metformin treatment are highlighted. Red and blue bars under each plot indicate females and males, respectively.

**Figure 3.**
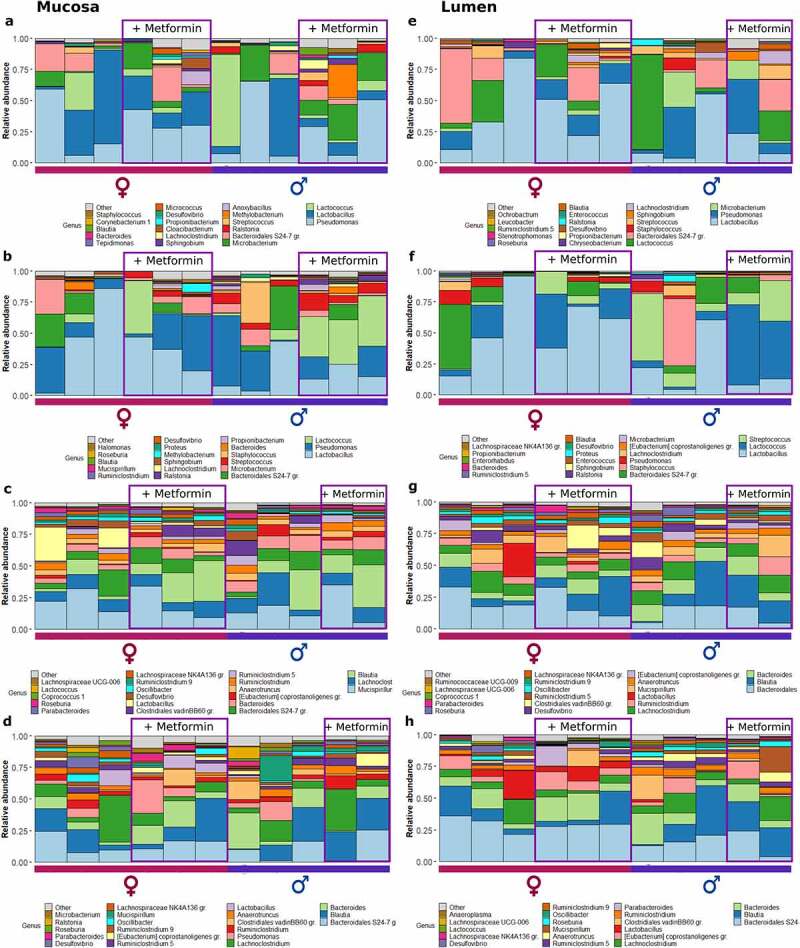
Microbiome composition at different sites of control diet-fed mice at the genus level – top 20 genera are shown. Mucosal layer: (a) proximal small intestine; (b) distal small intestine; (c) cecum; (d) colon. Luminal content layer: (e) proximal small intestine; (f) distal small intestine; (g) cecum; (h) colon. Samples representing mice receiving metformin treatment are highlighted. Red and blue bars under each plot indicate females and males, respectively.

To summarize the microbiome composition in each analyzed site, we compiled a list of the top 20 location-specific genera, as shown in Figures 2 and 3. We observed variation in microbial composition both longitudinally and cross-sectionally. Both parts of the small intestine were dominated by *Lactobacillus* in HFD-fed mice, followed by *Pseudomonas* and *Microbacterium* in the mucosal layer and *Pseudomonas* and *Lactococcus* in the luminal layer (Figure 2a,b,e and f). Likewise, *Lactobacillus* and *Pseudomonas* prevailed in CD-fed mice, followed by *Lactococcus* in the mucosal layer (Figure 3a,b). In the luminal layer, *Lactobacillus* was followed by *Pseudomonas* and *Microbacterium* in the proximal part of the small intestine and by *Lactococcus* and *Streptococcus* – in the distal part (Figure 3e,f). The relative abundance of *Lactococcus* was increased in the distal small intestine of CD-fed male mice receiving metformin treatment. In contrast, in HFD-fed mice, this effect was more pronounced in female mice in both parts of the small intestine. *Streptococcus* was more abundant in the small intestine of female HFD-fed mice than in males, and its relative abundance was higher in the metformin-treated animals.

In HFD-fed mice, *Blautia* had the highest relative abundance in the cecum at both layers (Figures 2c,g). *Mucispirillum*, *Lachnoclostridium*, and *Bacteroides* were among the other top genera in the cecum. In CD-fed mice, *Mucispirillum* showed the highest relative abundance in the mucosa, while luminal content was enriched in *Bacteroidales* representatives (Figures 3c,g). *Blautia* and *Lachnoclostridium* had high relative abundance in both layers. Similar to the cecum, in the colon of HFD-fed mice top bacteria were *Bacteroides* in the mucosa, followed by *Blautia* and *Lachnoclostridium*, and *Blautia* in the luminal layer, followed by *Bacteroides* and other *Bacteroidales* members (Figures 2d,h). Similar results were observed in CD-fed mice (Figures 3d,h).

### Alpha and beta diversity analysis

**Figure 4.**
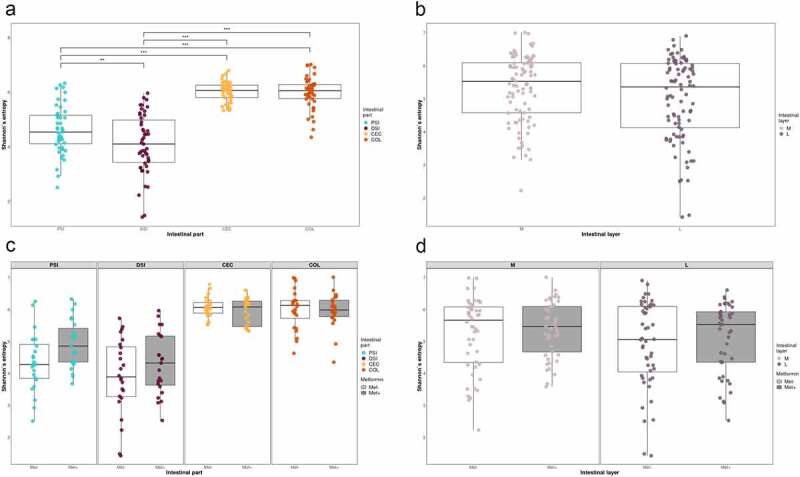
Microbiome alpha diversity analysis expressed as Shannon index: (a) between intestinal parts; (b) between intestinal layers. Microbiome alpha diversity analysis in response to metformin treatment expressed as Shannon index, *n* = 12 per group: (c) between intestinal parts; (d) between intestinal layers. PSI – proximal small intestine; DSI – distal small intestine; CEC – cecum; COL – colon; M – mucosa; L – lumen. **adjusted *p* < 0.05; *** adjusted *p* < 0.01.

Shannon diversity index analysis revealed significant differences in alpha diversity between all the studied intestinal segments, with the most pronounced differences present between the distal part of the small intestine and two other locations, cecum, H = 55.90, p-value<0.001 and large intestine, H = 55.67, p-value<0.001 (Figure 4). Similar results were observed for Pielou’s evenness and Faith’s phylogenetic diversity (data not shown). There were no significant differences in alpha diversity between intestinal layers for all the metrics analyzed. When the effect of metformin treatment on alpha diversity in each intestinal site was evaluated, we did not observe any significant differences after adjustment for multiple testing for all the metrics analyzed.

**Figure 5.**
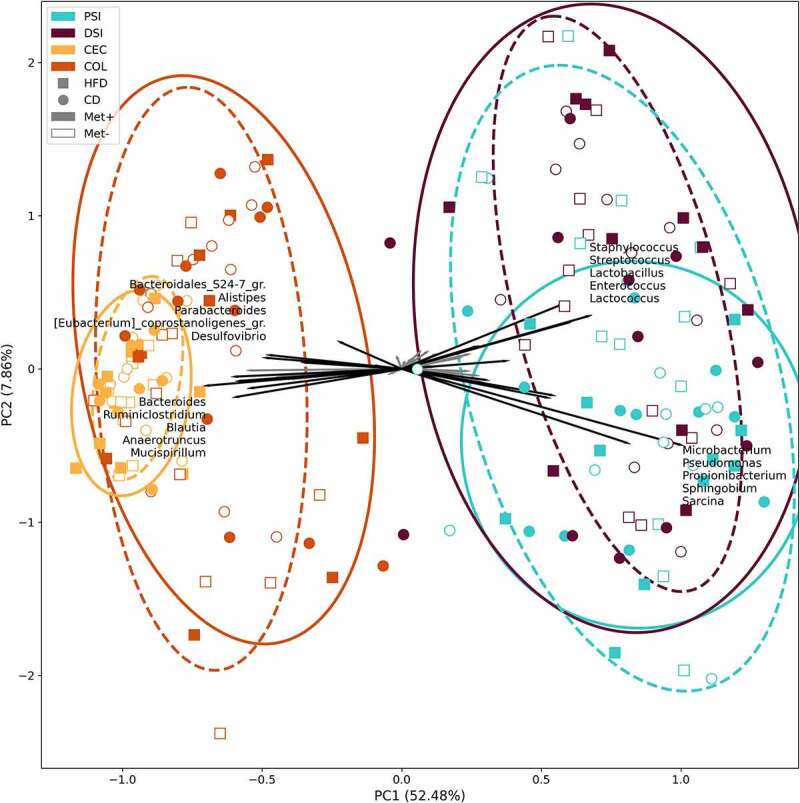
Beta diversity of all samples taken together was estimated using principal components analysis on centered log-ratio transformed values. Intestinal part, diet, and metformin treatment status are indicated. PSI – proximal small intestine; DSI – distal small intestine; CEC – cecum; COL – colon. In the corresponding intestinal part, continuous and dashed ellipses represent Met+ and Met- subsets, respectively.

**Figure 6.**
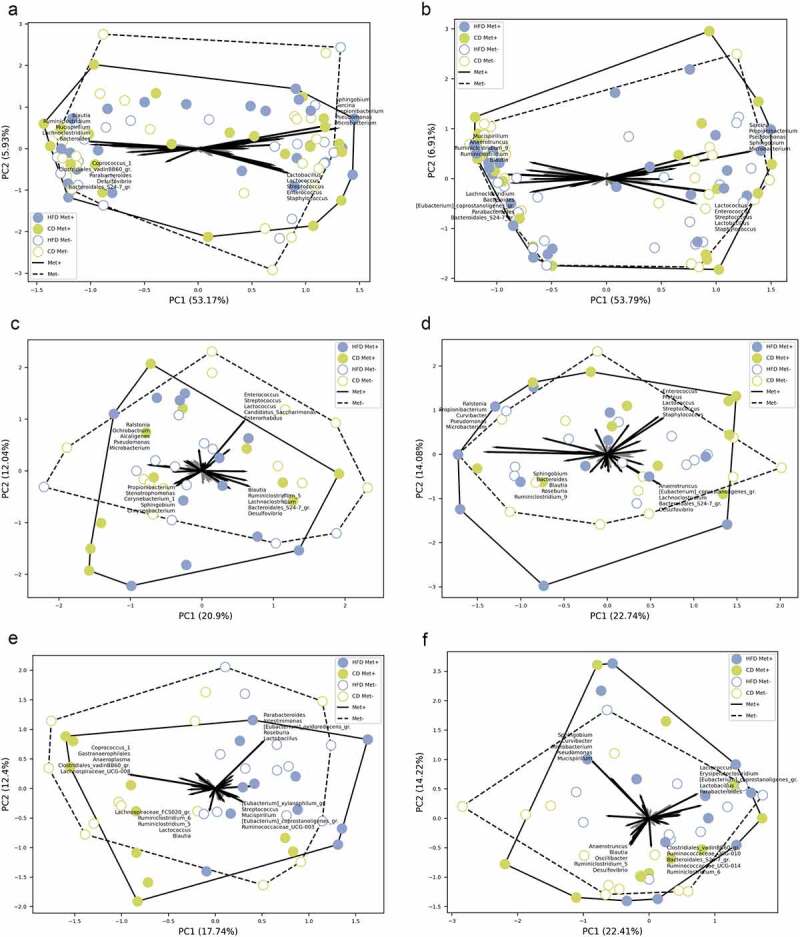
Beta diversity in each of the groups in response to 10 weeks long metformin treatment in each of the intestinal sites, *n* = 6 per group: (a) mucosa; (b) lumen; (c) proximal small intestine; (d) distal small intestine; (e) cecum; (f) colon.

Beta diversity was evaluated by ordination analysis. Biplots using PCA were generated, including each of the studied groups based on the diet type and metformin treatment status (Figures 5 and 6). When taken together, samples representing both parts of the small intestine clustered separately from the samples of the cecum and colon (Figure 5). *Sphingobium*, *Sarcina*, *Propionibacterium*, *Pseudomonas*, and *Microbacterium* representatives were the principal microbial identifiers of the proximal small intestine. *Lactococcus*, *Lactobacillus*, *Streptococcus*, *Enterococcus*, and *Staphylococcus* were the main drivers of the distal small intestine. In turn, cecum samples were identified by *Mucispirillum*, *Anaerotruncus*, *Blautia*, *Ruminiclostridium*, and *Bacteroides*. Colon was characterized by *Desulfovibrio*, *Parabacteroides*, *Eubacterium_coprostanoligenes_group* members, *Alistipes*, and *Bacteroidales_S24-7_group* (now known as *Muribaculaceae*) representatives.

A separate analysis of each intestinal part (Figure 6) revealed *Chryseobacterium*, *Propionibacterium*, *Corynebacterium_1*, and *Sphingobium* as the principal identifiers of metformin treatment in the proximal small intestine. The distal part was characterized by *Lactococcus*, *Streptococcus*, *Enterococcus*, *Proteus*, and *Staphylococcus* in CD-fed mice and *Blautia*, *Ruminiclostridium_9*, *Sphingobium*, *Bacteroides*, and *Roseburia* in HFD-fed mice. The main identifiers of metformin treatment in the cecum of HFD-fed mice were *Eubacterium* representatives, *Mucispirillum*, *Ruminococcaceae_UCG-003*, and *Streptococcus* and *Lachnospiraceae_FCS020_group*, *Ruminiclostridium* representatives, *Lactococcus*, and *Blautia* in CD-fed mice. At the same time, *Sphingobium*, *Curvibacter*, *Microbacterium*, *Pseudomonas*, and *Mucispirillum* were the strongest identifiers in the colon.

## Differentially abundant taxa

### Metformin treatment-mediated effects on the abundance of bacteria in different intestinal sites

**Figure 7.**
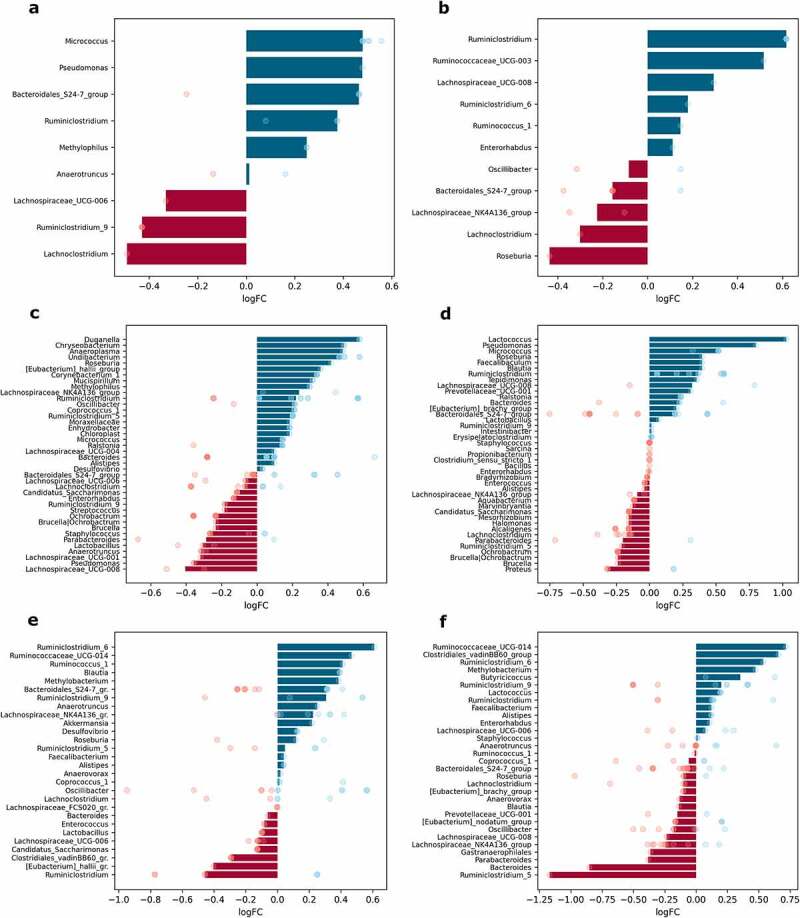
Differentially abundant genera in response to metformin treatment in different intestinal parts and layers (expressed as LogFC), *n* = 12 per group: (a) mucosa; (b) lumen; (c) proximal small intestine; (d) distal small intestine; (e) cecum; (f) colon. Blue bars represent genera with increased abundance among metformin users, and red bars – with decreased abundance. Dots of the corresponding color indicate all the individual features assigned to the genus.

When samples were contrasted regarding metformin treatment status (Met+ vs. Met- groups, *n* = 12 per group), up to 41 different genera were significantly differentially abundant in any of the parts of the small intestine; 28 genera in the cecum and 31 in the colon; and up to 11 genera in each of the intestinal layers studied (Figure 7).

Metformin treatment showed a varied effect in different sites of the intestine. The effect on the abundance of *Bacteroidales_S24-7_group* members was inversed in each layer – metformin increased the abundance of this genus in mucosa samples but decreased it in luminal content samples (Figure 7a,b). *Ruminiclostridium* increased in the mucosa and lumen, while *Lachnoclostridium* decreased in both layers. Several genera were significantly affected only in one of the layers. The abundance of *Roseburia* declined in luminal content samples. In contrast, the abundances of *Micrococcus*, *Pseudomonas*, and *Methylophilus* were significantly affected only in the mucosa.

Analysis of each of the intestinal parts separately revealed that metformin had a stronger effect on the abundance of the bacteria in the small intestine. Bacteria with increased abundances in response to metformin treatment only in the proximal small intestine include *Duganella*, *Chryseobacterium*, *Anaeroplasma*, *Undibacterium*, *Corynebacterium*, *Mucispirillum*, and *Methylophilus* (Figure 7c). In turn, *Eubacterium_halli_group* members were increased in the proximal small intestine but decreased in the cecum. Genera augmented uniquely in the distal part of the small intestine, include *Faecalibaculum* and *Tepidimonas*, while *Proteus* was depleted (Figure 7d). The abundance of *Roseburia* and *Micrococcus* was increased in both parts of the small intestine, while *Ochrobactrum* was decreased in these parts. *Pseudomonas* was affected in opposite directions in each part of the small intestine, with the abundance of the genus decreasing in the proximal part and increasing in the distal part. The most pronounced effect of metformin on the increased abundance was detected for *Lactococcus* in the distal small intestine; it was also increased in the colon, though to a lesser extent.

*Blautia* was enriched in the distal part of the small intestine and cecum but diminished in the colon samples. *Ruminiclostridium* increased in all sites except cecum, where it was one of the most depleted genera (Figure 7e). Several genera were altered in the cecum and colon in opposite directions. *Methylobacterium* and *Ruminococcaceae_UCG-014* were enriched in both parts, while *Bacteroides* was decreased. In turn, *Clostridiales_vadinBB60_group* members were decreased in the cecum and increased in the colon. *Butyricicoccus* was significantly increased uniquely in the colon, but *Gastranaerophilales* was decreased (Figure 7f). *Ruminiclostridium_5* was the most reduced genus in response to metformin treatment in the colon, but it was increased in the proximal small intestine. The abundance of *Parabacteroides* was decreased in both parts of the small intestine and colon but was not affected in the cecum. A similar pattern was detected for *Lachnospiraceae_UCG-008*, with the only difference being that it was augmented in the distal part of the small intestine.

### Sex-related differences in the abundance of microbiome members in the intestinal sites studied

**Figure 8.**
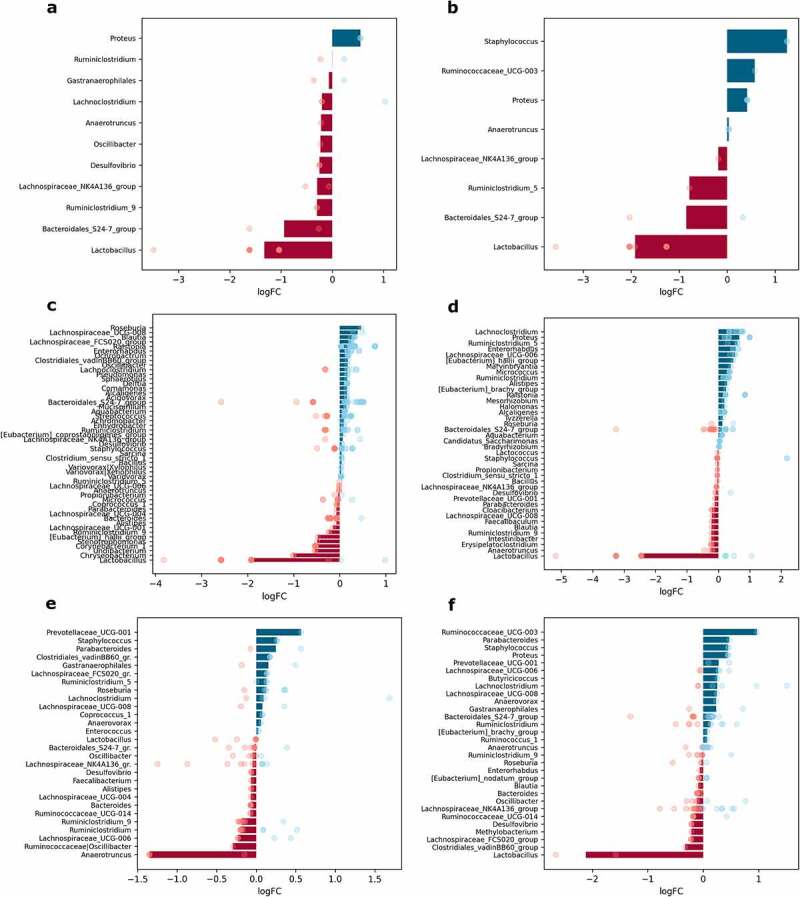
Differentially abundant genera between sexes in different intestinal parts and layers (expressed as LogFC), *n* = 12 per group: (a) mucosa; (b) lumen; (c) proximal small intestine; (d) distal small intestine; (e) cecum; (f) colon. Blue bars represent genera with increased abundance among males, and red bars – with decreased abundance. Dots of the corresponding color indicate all the individual features assigned to the genus.

The effect of sex was evaluated by contrasting the samples from male mice of all experimental groups with corresponding samples from female mice at each intestinal site separately, *n* = 12 per group. In total, 11 genera in mucosal samples and 8 genera in luminal content samples were differentially abundant between males and females. When each of the intestinal parts was analyzed separately, 51 genera in the proximal small intestine; 40 genera in the distal small intestine; 28 genera in the cecum; and 30 genera in the colon were significantly differentially abundant between sexes (Figure 8).

*Lactobacillus* showed the most pronounced differences between sexes, with being decreased in males both in mucosal and luminal content samples (Figures 8a,b). Members of the *Bacteroidales_S24-7_group* were also depleted in both layers in males compared to females. In contrast, *Proteus* was increased in males in both mucosa and lumen, while *Staphylococcus* and *Ruminococcaceae_UCG-003* were increased, and *Ruminiclostridium_5* was decreased only in the lumen.

The abundance of *Lactobacillus* was strongly reduced in all of the intestinal parts of males (distinct features in the cecum). Among the genera with significantly different abundance only in the proximal small intestine, *Roseburia* was enriched, while *Chryseobacterium*, *Undibacterium*, and *Corynebacterium* were depleted in males relative to females (Figure 8c). *[Eubacterium]_hallii_group* was affected by the sex in the small intestine – in males, it was decreased in the proximal part but increased in the distal part of the small intestine; however, *Enterorhabdus* was enriched in both parts. *Lachnoclostridium* and *Ruminiclostridium_5* were increased in the distal part of the small intestine in males compared to females (Figure 8d). Meanwhile, the abundance of *Proteus* was increased in both the distal small intestine and colon. The abundance of *Anaerotruncus* was markedly decreased in the cecum of males (Figure 8e). *Ruminococcaceae_UCG-003* was increased in males only in the colon. *Parabacteroides*, *Staphylococcus*, and *Prevotellaceae_UCG-001* representatives were enriched in the cecum and colon in males compared to females (Figure 8e,f).

### Diet-induced effects on the abundance of intestinal microbiome representatives

**Figure 9.**
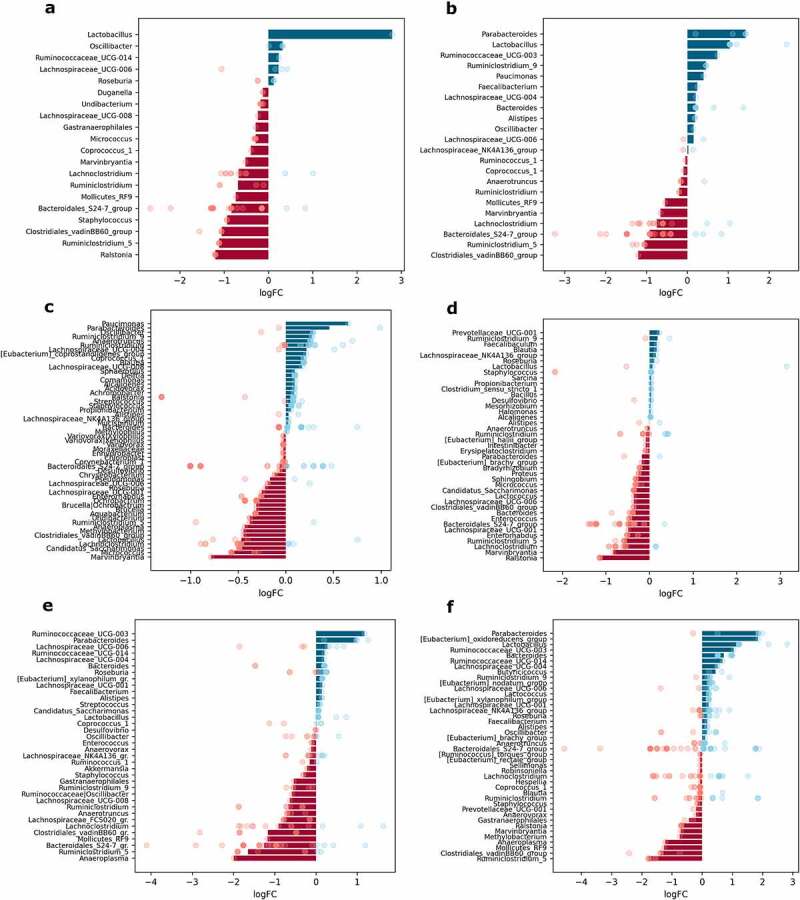
Differentially abundant genera between high-fat diet-fed and control diet-fed mice in different intestinal parts and layers (expressed as LogFC), *n* = 12 per group: (a) mucosa; (b) lumen; (c) proximal small intestine; (d) distal small intestine; (e) cecum; (f) colon. Blue bars represent genera with increased abundance among HFD-fed mice, and red bars – with decreased abundance. Dots of the corresponding color indicate all the individual features assigned to the genus.

To assess dietary effects, samples from all HFD-fed mice were compared with corresponding samples from CD-fed mice at each intestinal site separately, *n* = 12 per group. Diet significantly affected the abundance of 20 genera in the mucosa, 22 genera in the lumen, 55 and 41 genera in the proximal and distal small intestine, respectively, 36 genera in the cecum, and 42 genera in the colon (Figure 9).

The abundance of *Lactobacillus* increased in HFD-fed mice in both studied layers LogFC = 2.79 ± 0.48, FDR<0.001 and LogFC = 2.44 ± 0.53, FDR = 0.002 for mucosa and lumen, respectively (Figures 9a,b). In contrast, *Ruminiclostridium_5*, *Mollicutes_RF9*, *Lachnoclostridium*, *Marvinbryantia*, and *Clostridiales_vadinBB60_group* members were significantly lowered in both layers in response to HFD feeding. *Ralstonia* and *Staphylococcus* were decreased in HFD-fed mice solely in mucosa samples. *Parabacteroides* and *Ruminiclostridium_9* were significantly increased in the lumen of HFD-fed mice exclusively.

Uniquely to the proximal small intestine, the abundance of *Paucimonas* and *Oscillibacter* increased, while *Undibacterium* decreased in HFD-fed mice (Figure 9c). Genera, which significantly decreased in both parts of the small intestine in response to HFD feeding, include *Micrococcus*, *Enterorhabdus*, and *Candidatus_Saccharimonas* (Figures 9c,d). *Staphylococcus* was depleted in the cecum of HFD-fed mice (Figure 9e). In turn, *Eubacterium_oxidoreducens_group* members were increased only in the colon (Figure 9F). *Mollicutes_RF9* was decreased in both the cecum and colon of HFD-fed mice, while *Ruminococcaceae_UCG-003* was increased in these parts. In HFD-fed mice, representatives of two genera, *Ruminiclostridium_5* and *Clostridiales_vadin_BB60_group*, were decreased in all intestinal parts compared to CD-fed mice. As described above, these genera were also depleted in both intestinal layers of HFD-fed mice.

### Differentially abundant bacteria between the mucosa and the lumen in all intestinal parts

**Figure 10.**
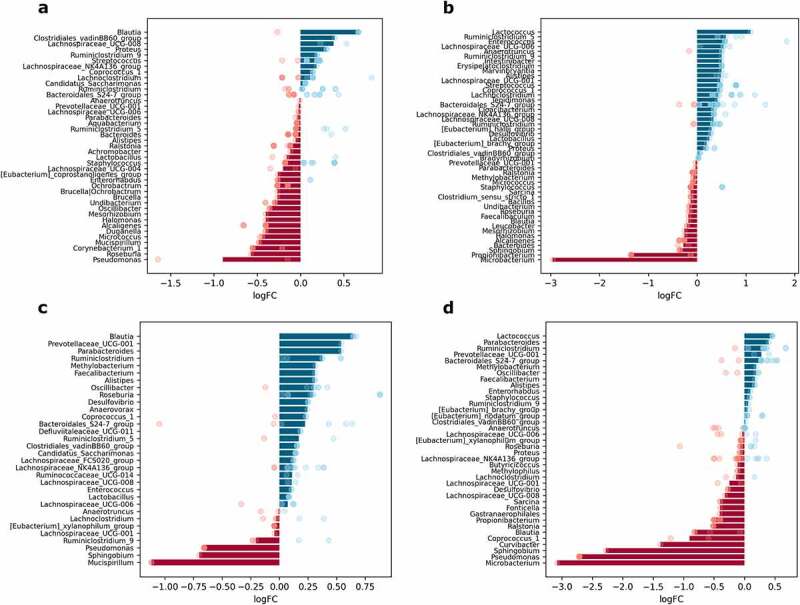
Differentially abundant genera between lumen and mucosa in different intestinal parts (expressed as LogFC), *n* = 12 per group: (a) proximal small intestine; (b) distal small intestine; (c) cecum; (d) colon. Blue bars represent genera with increased abundance in the lumen, and red bars – with decreased abundance. Dots of the corresponding color indicate all the individual features assigned to the genus.

The luminal and mucosal samples from all experimental groups were contrasted in each of the intestinal parts to investigate layer-related differences in microbiome composition, *n* = 12 per group. A total of 41 genera in the proximal small intestine, 48 – in the distal small intestine, 32 – in the cecum, and 38 – in the colon were significantly differentially abundant between the lumen and mucosa (Figure 10).

Differential abundance analysis between mucosa and lumen layers in each of the intestinal parts separately revealed substantial spatial variation of genera. Uniquely to the proximal small intestine, its lumen was depleted of *Corynebacterium_1* and *Micrococcus* (Figure 10a). Genera specifically increased in the lumen of the distal part of the small intestine include *Erysipelatoclostridium*, *Intestinibacter*, and *Marvinbryantia*. *Anaerotruncus* and *Lachnospiraceae_UCG-006* also were enriched in the lumen of the distal small intestine (Figure 10b).

Several genera were increased in the cecum and colon, including *Parabacteroides*, *Prevotellaceae_UCG-001*, *Methylobacterium*, and *Faecalibacterium* (Figures 10c,d). The abundance of *Mucispirillum* was lower in the lumen of the proximal small intestine and cecum. Similarly, *Oscillibacter* was reduced in the lumen of the proximal small intestine but increased in the cecum. *Ruminiclostridium_5* and *Enterococcus* were enriched in the lumen of the distal small intestine and cecum. Genera with altered abundance, specifically in the distal small intestine and colon, include *Microbacterium* and *Propionibacterium*, with reduced abundance in the lumen of both parts, and *Lactococcus*, with increased abundance in the same sites.

*Curvibacter* was depleted solely in the lumen of the colon. *Roseburia* and *Ruminiclostridium_9* were altered in opposite directions in the same sites. *Roseburia* was decreased in both parts of the small intestine and increased in the cecum; in turn, *Ruminiclostridium_9* was depleted in the small intestine and enriched in the cecum. *Ruminiclostridium*, together with *Alistipes* and *Sphigobium*, were oppositely affected, the former two being enriched in the lumen of the distal small intestine, cecum, and colon, while the latter was reduced in the same sites. *Coprococcus_1* and *Blautia* were differentially abundant between layers in all intestinal parts. *Blautia* was increased in the lumen of the proximal small intestine and cecum but decreased in the distal part of the small intestine and colon. *Coprococcus_1* was enriched in the lumen in all parts of the intestine except the colon, where the abundance of the genus was reduced.

### Interaction of metformin treatment with diet type, sex, and intestinal layer

**Figure 11.**
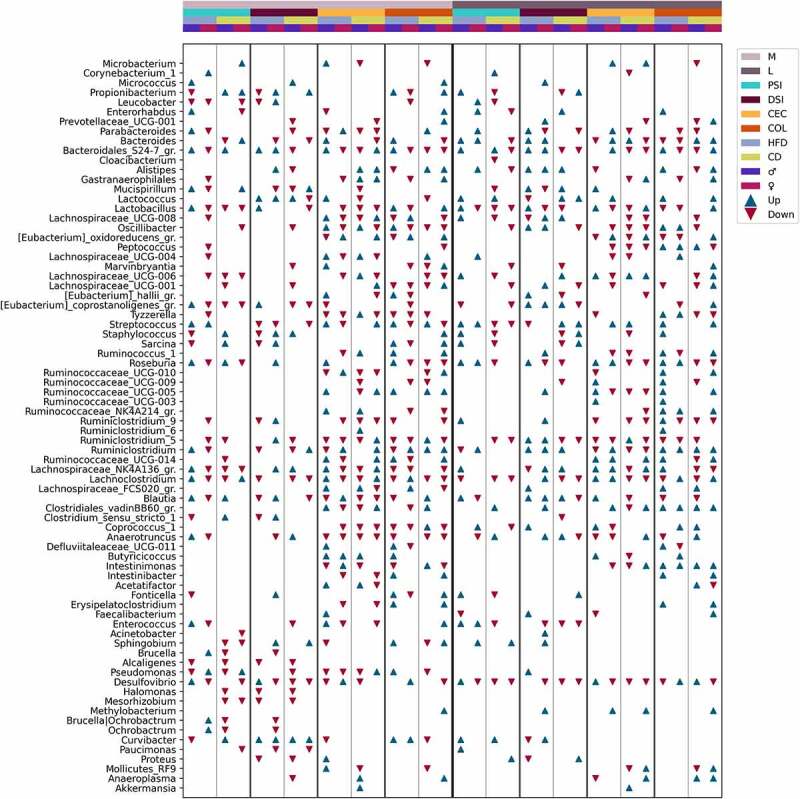
Summary of differentially abundant genera between Met+ and Met- mice in different subsets formed by various combinations of the levels of studied factors (expressed as LogFC), *n* = 3 per group. Only the genera with an absolute LogFC≥1 in at least one of the subsets are included, indicating the directions of changes (with an absolute LogFC>0.2), if detected, in all subsets. M – mucosa; L – lumen; PSI – proximal small intestine; DSI – distal small intestine; CEC – cecum; COL – colon. Blue triangle – an increase of the abundance among metformin users; red triangle – a decrease among metformin users.

The genera with the most substantial differences in response to metformin treatment in each of the analyzed subsets are summarized in Figure 11. In total, we found 77 genera with a LogFC of at least 1 in any of the analysis subsets (Figure 11). Complete lists of statistically significantly altered genera in response to metformin treatment in each subset are shown in Supplementary Figures S1–S4. Metformin affected representatives of all the main phyla found in the intestine – *Bacteroidetes*, *Firmicutes*, *Actinobacteria*, *Proteobacteria*, and *Verrucomicrobia*.

Members of *Betaproteobacteria*: *Paucimonas* and *Alcaligenes* were reduced only in the mucosal layer of both parts of the small intestine, while another representative *Curvibacter* was altered in opposite directions in different sites, including the cecum and colon in the mucosal layer but not in the luminal layer. *Deltaproteobacteria* member *Desulfovibrio* was affected by metformin almost in all studied sites. The most remarkable changes in abundance were observed in the proximal small intestine in both layers and the distal small intestine in the mucosal layer of HFD-fed mice. In addition, this genus showed marked sexual dimorphism in response to metformin treatment, with being increased in HFD-fed males in both parts of the small intestine and decreased in females in the mucosal layer and the proximal small intestine and cecum in the luminal layer. In CD-fed mice, *Desulfovibrio* was reduced in both sexes, though it was more pronounced in females. Sex-related differences in the proximal small intestine were also observed for *Pseudomonas* in mice fed both diet types, with being reduced in males and increased in females. The abundance of *Pseudomonas* was altered only in the mucosal layer, except for an increase in the lumen of the distal small intestine of HFD-fed males.

The most pronounced changes in the abundance of *Actinobacteria* members were found in both layers of the proximal small intestine; however, significant changes in at least one genus were observed in all intestinal parts. *Bacteroides* was increased in both sexes of HFD-fed mice in the lumen of both parts of the small intestine and decreased in the colon. In turn, in the colon of CD-fed mice, *Bacteroides* was increased. *Bacteroidales_S24–7* group (*Muribaculaceae*) was altered in almost all studied sites. The strongest reduction in the abundance of the genus was found in the mucosa of the distal small intestine of CD-fed males.

The abundance of *Akkermansia* was significantly affected exclusively in CD-fed male cecum in both layers. *Mucispirillum* was strongly decreased in the mucosa of the proximal and distal small intestine of HFD-fed females, while it was slightly increased in males. In contrast, CD-fed females had marked increases in the genus at the exact locations. In the luminal layer, *Mucispirillum* was depleted in the proximal small intestine of CD-fed males and the distal small intestine of HFD-fed females, whereas the genus was increased in the distal small intestine of males fed both diet types and in HFD-fed males in the cecum.

A substantial interaction between metformin treatment and sex, diet type, and the intestinal layer was observed regarding the abundance pattern of *Bacilli* members. *Lactobacillus*, *Enterococcus*, *Staphylococcus*, and *Streptococcus*, opposite to *Lactococcus*, were markedly decreased in the lumen of the proximal small intestine of CD-fed males. In contrast, all genera together with *Lactococcus* were increased in HFD-fed males at the same site. *Lactococcus* was substantially increased in the distal small intestine of CD-fed mice of both sexes, while in HFD-fed males, it was reduced. *Lactococcus* was increased in HFD-fed males and CD-fed females in the lumen of the colon. In the mucosa, the abundance of *Lactococcus* was not affected by metformin in the proximal small intestine. However, it was increased in both sexes of HFD-fed mice and CD-fed males in the distal small intestine, whereas in females, it was reduced. The abundance of *Lactobacillus* was mainly reduced in response to metformin, though it was augmented in the mucosa of the proximal small intestine of HFD-fed males.

*Intestinimonas* and *Clostridiales_vadinBB60_group* members were affected only in the cecum and colon in the mucosa layer. A similar pattern was observed in the lumen, except that *Clostridiales_vadinBB60_group* was strongly increased in CD-fed males in the proximal small intestine and HFD-fed females in the distal small intestine. *Butyricicoccus* was affected similarly, mainly being augmented only in the mucosa of the cecum of male mice and the colon of HFD-fed males. In the lumen of the cecum, HFD-fed male mice were enriched in *Butyricicoccus*, while in CD-fed males genus was reduced. *Butyricicoccus* was increased in HFD-fed females in the lumen of the colon but unchanged in other subsets. Other *Clostridiaceae* representatives were not affected in the cecum and colon in the luminal layer.

Genera representing families *Lachnospiraceae*, *Peptococcaceae*, *Peptostreptococcaceae*, and *Ruminococcaceae* were affected more in the cecum and colon in both layers. In contrast to this observation, *Eubacterium_coprostanoligenes_group* members were more affected in both parts of the small intestine in both layers. In general, members of the families mentioned above were enriched in the mucosa of the proximal small intestine of HFD-fed males but decreased in HFD-fed females. Other features common to these genera (with a few exceptions) are a decrease in the lumen of the proximal small intestine and cecum of CD-fed mice; and enrichment in the distal small intestine and colon (predominantly males) of HFD-fed mice. *Roseburia* showed strong sex-related differences in the abundance changes in response to metformin treatment. The abundance of the genus was increased in the mucosa of the proximal small intestine of male mice fed both types of diet and decreased in females. *Roseburia* was enriched only in HFD-fed males in the cecum but unchanged in other subsets. Similarly, it was increased in the colon of HFD-fed males, and a decrease was also found in other subsets, CD-fed mice and HFD-fed females. In the lumen, *Roseburia* was increased in HFD-fed mice of both sexes in all intestinal parts (except a decrease in females in the distal small intestine) and decreased in CD-fed mice of both sexes in the cecum and colon and both parts of the small intestine in males only.

## Discussion

The effect of metformin therapy on the composition and function of the gut microbiome has been demonstrated in previous studies;^20–26^⁠⁠ however, an in-depth analysis of spatial variation of the effects of long-term metformin treatment on the gastrointestinal tract has not yet been performed in vivo. Our study provides novel information on the effects of metformin on both the gastrointestinal mucosa and the luminal layers at four different sites of the gut. Furthermore, our animal experiment included both sexes of mice, thus enabling an additional dimension of analysis of metformin’s effects. Finally, the metformin dosage was calculated to correspond to the therapeutic dose in humans, which makes our study more clinically relevant than experiments in which metformin was administered at the supratherapeutic level.

We observed significantly different alpha diversities between all the studied intestinal parts corresponding to anatomical and functional differences of the various sites. The cecum and colon microbiomes were more diverse than those of the small intestine, consistent with a previous report.^27^⁠ The relative abundance of *Lactobacillus* decreased along the intestinal tract toward the colon, as described before.^9^⁠⁠⁠ Beta diversity analysis showed a clear difference between microbial communities of the small intestine and the cecum and colon, similar to a previous study comparing different parts of the gastrointestinal tract of male mice.^27^⁠ Analysis revealed that metformin treatment has the most pronounced effect on the samples representing the proximal small intestine, followed by the distal small intestine. Cecum samples of all experimental groups clustered most closely, showing the least effect of any studied factors on this intestinal part and suggesting its relative stability.

We have shown the effect of metformin treatment, sex, diet type, and intestinal layer on the spatial variation of the gut microbiome by analyzing each of these factors separately. Furthermore, we have investigated the effect of metformin treatment in each of the subsets formed by combinations of levels of the studied factors: intestinal layer, intestinal part, diet type, and sex, and detected substantial variation of metformin’s effects in each of these subsets.

Metformin treatment had a more pronounced effect on the microbiome composition in both parts of the small intestine (indicated by higher absolute LogFC values). This finding confirms the hypothesis that the effect of metformin treatment is not uniform in the whole intestine but rather depends on the absorption characteristics of the medication. When all mice were contrasted based on metformin treatment status, several genera were increased uniquely in the proximal small intestine, where according to a previous study,^16^⁠ the absorption of metformin occurs most. Among these genera, many are aerobic bacteria – *Duganella*, *Chryseobacterium*, *Undibacterium*, *Corynebacterium*, *Methylophilus*, and the anaerobes *Anaeroplasma* and *Mucispirillum*. *Mucispirillum* is commensal in the microbiota of humans and various vertebrates. The only species of the genus, *Mucispirillum schaedleri*, is a core member of the laboratory mouse microbiota throughout the whole gastrointestinal tract. *Mucispirillum schaedleri* has not been widely identified in human studies due to its low abundance in fecal samples, but it is enriched in the intestinal mucosa.^28^⁠⁠ Genomic data have indicated that instead of degrading mucosal glycans such as mucin, *M. schaedleri* predominantly processes monosaccharides, oligopeptides, amino acids, glycerol, and short-chain fatty acids produced by other bacteria.^29^⁠⁠

Metformin treatment substantially increased the abundance of *Lactococcus* in the distal part of the small intestine. This is consistent with a previous study with a similar duration and route of metformin treatment but using only aged male mice, where an increase in *Lactococcus* was observed in fecal samples after treatment with metformin.^23^⁠ Lactic acid bacteria, including *Lactococcus*, produce lactate, a substrate for other members of the microbiota to convert it into butyrate.^8^⁠ We observed a subtle increase in another lactate producer, *Lactobacillus*, in the distal small intestine, whereas the abundance of the genus was reduced in the proximal small intestine and cecum and unchanged in the colon. Previous studies^24,30^⁠ have produced conflicting results regarding the effect of metformin on *Lactobacillus*.^31^⁠ This could be explained by using fecal samples that do not fully represent the small intestinal microbiota, differences in experimental designs, and species-specific effects not detected in the genus-level analysis.^32^⁠ This together with the intestinal part-unique effects of metformin treatment, further supports the proximal and the distal part of the small intestine as principal sites of metformin-mediated effects on the gut microbiome.⁠⁠⁠⁠

Our results show that metformin mainly targets genera belonging to *Clostridia* in all intestinal sites. Altered genera include the representatives of *Lachnospiraceae* (*Blautia*, *Lachnoclostridium*, *Lachnospira*, *Marvinbryantia*, *Roseburia*), *Clostridiaceae* (*Butyricicoccus*, *Clostridium*), *Ruminococcaceae* (*Anaerotruncus*, *Faecalibacterium*, *Oscillibacter*, *Ruminococcus*), and *Eubacteriaceae* (*Eubacterium*). This is consistent with a similar study in which the effect of short-term metformin treatment on the composition of the microbiome representing the intestinal lumen at different sites was assessed in male mice, showing a reduction of *Clostridium* and *Lachnospiraceae* members in response to metformin treatment.^19^⁠ We found an increase of *Roseburia* in both parts of the small intestine and depletion of the genus in the cecum and colon in the luminal layer. This could explain the previously described reduced abundance of *Roseburia* in the feces of HFD-fed mice after metformin treatment,^23^⁠ as feces mostly correspond to the samples of colonic contents.

In addition, we observed a strong sex-specific effect on the spatial variation of the gut microbiome when all mice were contrasted based on sex, as the abundance of *Lactobacillus* was strongly reduced in all intestinal parts and layers of males. This finding is supported by a study in healthy humans, where an increase of *Lactobacillales* in female mucosa-associated microbiota was reported.^33^⁠⁠ *Lactobacillus* has been shown to be increased in diabetes patients and animals fed with a high-fat diet,^2,13,34^⁠ consistent with enrichment of the genus in the colon and both intestinal layers of HFD-fed mice compared to CD-fed mice in our study. However, we observed that *Lactobacillus* was decreased in the proximal small intestine of HFD-fed mice, showing the differences between the composition of the fecal microbiome and the microbiome of different intestinal parts. Previous studies have shown that *Lactobacillus* spp. reduce blood glucose levels in high-fat diet-induced diabetic male mice;^35^⁠⁠ however, these are positively correlated with blood glucose levels in European women with T2D,^36^⁠ and metabolites produced by *Lactobacilli* may contribute to the glucose modulation of metformin.^37^⁠⁠ Sex-related differences in *Lactobacillus* abundance that we observed might partially explain these discrepancies emphasizing the need to perform studies on individuals of both sexes to obtain as complete information as possible. Additionally, an increase in *Anaerotruncus* has been found in women with metabolic syndrome compared to men.^38^⁠ Our data support these sex-related differences, narrowing them down to the cecum. We also detected a higher abundance of opportunistic pathogens *Proteus* in both layers and *Staphylococcus* in the lumen of male mice compared to females. *Proteus* was increased in the distal small intestine, *Staphylococcus* in the cecum, and both genera in the colon of males. This suggests that the microbiota of male mice in this model contains more opportunistic pathogens than females, which could contribute to another layer of phenotypic sexual dimorphism reported in HFD-fed mice.^39^

The role of other factors not analyzed in this study, including sex hormones, stress, dietary fiber content affecting systemic estrogen levels, the interaction between sex and genotype, and circadian rhythms, also might be driving the sex-related differences in the gut microbiome.^40–42^⁠ In addition, macronutrient levels and diet composition, including the availability of short-chain fatty acids, contribute to the effect of diet.⁠⁠^43^⁠ Wang et al. compared microbiome composition between HFD-fed and low-fat diet-fed mice in the cecum and colon and, similar to our results, found a higher abundance of *Lachnospiraceae_UCG-006* in the cecum of HFD-fed mice.^13^⁠ We, however, add that in both parts of the small intestine, *Lachnospiraceae_UCG-006* is reduced in HFD-fed mice, indicating that the effect of HFD feeding on this genus is not uniform throughout the intestine. Furthermore, we did not observe significant differences in the abundance of *Blautia* in the cecum, which was reported to be increased in this intestinal part, although it was enriched in both parts of the small intestine in our data. ⁠

Analysis between the lumen and mucosa in each of the intestinal parts revealed that *Intestinibacter*, *Erysipelatoclostridium*, and *Marvinbryantia* are genera enriched in the lumen of the distal small intestine uniquely. All these genera have not been extensively studied; *Intestinibacter* has been suggested to be involved in mucus degradation;^2^⁠⁠ a representative of *Erysipelatoclostridium* (formerly named *Clostridium ramosum*) has been associated with diet-induced obesity in gnotobiotic mice;^44^⁠ *Marvinbryantia* has been described as fermenting glucose to acetate in the presence of high formate concentrations.^45^⁠ Enrichment of *Prevotellaceae_UCG-001*, *Oscillibacter*, and *Parabacteroides* in the lumen of the cecum and colon agrees with a study comparing lumen-associated and mucosa-associated microbiota.^33^⁠

⁠We observed an interaction between metformin treatment and each of the levels of studied factors, showing the complex landscape of metformin’s effects on the gut microbiome. Many genera were strongly affected in only one of the sexes or specific intestinal sites. For example, this analysis allowed us to confirm the promoting effect of metformin on the abundance of *Lactococcus* in the distal small intestine while adding information that it is mainly confined to the mucosa and that the abundance of *Lactococcus* is reduced in the lumen of HFD-fed males. In addition, we found that the genus is increased in the proximal small intestine of male mice only in the lumen.

This study has some limitations. First, although we evaluated the appropriate sample size before conducting the experiment, the study would have benefited from a larger sample size for each group studied. Second, due to the complexity of the Sidle algorithm, we had to balance the sampling depth and the number of samples still available after sampling, resulting in a lower resolution for taxa with small sequence counts to retain the statistical power. Finally, we observed considerable variability in gut microbiome composition between cages, so future studies should carefully consider the appropriate experimental unit for such studies.

In conclusion, our study describes the spatial differences of the metformin’s effects on the gut microbiome in luminal and mucosal layers of the intestine using both sexes of mice. Our results have revealed that metformin mainly exerts its microbiome-modulating effects in the small intestine, increasing the abundance of *Lactococcus* among other bacteria, while the impact on the microbiome of the cecum and colon is less pronounced. The effect of metformin on microbiome composition depends on sex, diet type, and intestinal layer. We furthermore emphasize the importance of including animals of both sexes in studies investigating the effects of metformin on the gut microbiome.

## Materials and methods

### Ethics statement

Animal procedures were reviewed and approved by the National animal welfare and ethics committee (Permit No. 91).

### Animals, study design, and procedures

The animal experiment has been described previously.^46^⁠ In short, C57BL/6N mice of both sexes with SPF status were purchased from the University of Tartu Laboratory Animal Centre and adapted to the local animal facility for one week. All the animals were housed under SPF conditions, 23 ± 2 ºC, with 55% humidity. The light cycle was 12:12 hours, with a light period from 7:00 am to 7:00 pm. Animals were housed in individually ventilated cages (Tecniplast) of up to three same-sex animals per cage on aspen bedding mixed with ALPHA-dri. Mice were fed HFD (rodent diet with 60 kcal% fat (D12492, Research Diets)) or CD (rodent diet with 10 kcal% fat (D12450J, Research Diets)) *ad libitum* and had free access to drinking water. At the initiation of the study, all mice were six weeks old. The total number of animals was 72, forming 24 experimental units – the cages with animals. For further analysis, each experimental unit was represented by one animal from the corresponding cage. The sample size was determined by the resource equation method, which is suitable for complex designs.^47^⁠

The study had a randomized block design and included eight experimental groups – HFD_M_Met-, HFD_F_Met-, HFD_M_Met+, HFD_F_Met+, CD_M_Met-, CD_F_Met-, CD_M_Met+, and CD_F_Met+, depending on T2D status induced by high-fat diet (HFD) or control diet (CD) feeding; sex (M or F for males and females respectively); and metformin treatment status (Met+ and Met- indicating exposure to metformin treatment) (Figure 1). Type 2 diabetes induction and biochemical parameters were described previously^46^⁠⁠ in detail. The duration of type 2 diabetes induction with HFD feeding was 20 weeks. Diagnostic criteria for T2D included increased body weight, increased fasting plasma glucose and insulin levels in HFD-fed mice compared to CD-fed mice, and a HOMA-IR index of at least 2. Metformin was then administered in the drinking water at a concentration calculated to correspond to 50 mg/kg body mass/day for 10 weeks while continuing to be fed either an HFD or CD. During the treatment period, all bottles, including those of the control group, were changed daily, and metformin was added freshly to the drinking water of the treatment groups.

### Sample collection

All the animals were sacrificed by cervical dislocation. Luminal and mucosal microbiome samples representing four different intestinal segments – proximal small intestine (duodenum-jejunum), distal small intestine (ileum), cecum, and colon were collected (Figure 1). First, intestinal segments were rinsed separately with distilled water to collect luminal contents. Second, rinsed tissue samples were put in a tissue dish containing cold PBS and cut longitudinally. Third, the remaining lumen contents were removed by repeated rinsing in separate clean tissue dishes. Finally, mucosa samples containing microbiome were obtained by scraping the inner intestinal surface of the intestinal segment with a cell scraper and collected in sterile tubes. Samples were stored at 80ºC until further analysis.

### DNA isolation and 16S rRNA gene amplification

Bacterial DNA was isolated from the collected samples using FastDNA Spin Kit for Soil (MP Biomedicals) according to the manufacturer’s instructions, and the concentration was measured using the Qubit 2.0 Fluorometer (Invitrogen). The concentration of DNA before library preparation was normalized to 5 ng/µl for every sample. The V1-V2, V3-V4, and V5-V6 hypervariable regions of the 16S rRNA gene were amplified by PCR using specific primers (Supplementary Table 1) tagged with Illumina sequencing adapters and sample-specific barcodes according to Illumina’s instructions. PCR products were analyzed by 1.2% agarose gel electrophoresis and purified using NucleoMag (Macherey-Nagel) magnetic beads. Purified amplicons were pooled at equimolar concentrations, and sample indexes were added by additional PCR. The quality of libraries was assessed by Agilent High Sensitivity DNA Kit (Agilent Technologies) on the Agilent 2100 Bioanalyzer (Agilent Technologies). Samples were sequenced on Illumina MiSeq (Illumina) platform with MiSeq Reagent Kit V2 (500-cycles) (Illumina) according to the manufacturer’s instructions.

### Data analysis

Data analysis began with evaluating sequencing data quality and read count distribution per sample group using the FastQC (v0.11.9)^48^⁠⁠ and MultiQC (v1.12)⁠^49^⁠ tools to identify possible sample level outliers and adapter contamination. Most of the analysis and diversity index calculation was performed using QIIME2 (v2022.2)⁠^50^⁠ microbiome analysis environment. First, we processed the regional amplicons for each individual, using the Cutadapt plugin^51^⁠ to trim the forward and reverse primers for each amplicon, specifying the allowed error rate of 0.1 and allowing for indels or deletions in bases when matching with the primers. In this step, reads that did not match the primers were discarded.

The demultiplexed regional sequences were denoised with the DADA2^52^⁠⁠ plugin to generate Amplicon Sequence Variants (ASVs). The trim lengths for DADA2 were selected for each region based on the base quality drop-off threshold from the visual inspection of the sample group level sequence quality box and whiskers plots to maximize the number of merged reads (Suplementary Table 1). Subsequently, all merged reads for each region were truncated to the length of 200 base pairs. The SILVA (v128) taxonomic database^53^⁠ was imported into QIIME 2 using the RESCRIPt⁠^54^⁠ plugin; the database was filtered to remove any sequence with more than 5 degenerate nucleotides. Regional database reads were extracted using the q2-feature-classifier plugin.^55^⁠ The regional database reads were aligned with the representative ASV sequences, allowing a mismatch of 2 nucleotides. Regional average relative abundances were solved through the Sidle implementation of the Short Multiple Reads Framework (SMURF) algorithm.^56,57^⁠ The phylogenetic tree was reconstructed by inserting consensus sequences for reconstructed amplicons into the SILVA (v128) backbone using the SEPP algorithm^58^⁠⁠ phylogenetic reference backbone while also inserting sequences that did not align with the SILVA taxonomy reference database. To discard low information or artifact sequences, the reconstructed ASV table was frequency filtered for features observed in at least three samples and with a taxonomic classification of genus level or higher. From the resulting table, a random feature subsample of 945 sequences per sample was made to normalize for the differences in library size, which was then used to calculate Shannon diversity, Faith’s phylogenetic diversity, and Pielou’s evenness alpha diversity indices.

Statistically significant differences in the alpha diversity between analyzed groups were identified using the Wilcoxon Rank Sum test and Benjamini-Hochgberg’s procedure. False discovery rate (FDR) values<0.05 were considered statistically significant. Further analysis and visualization of results were performed in the RStudio (v2021.09.0) environment. Reconstructed and classified data was then imported into the phyloseq (v1.38.0)^59^⁠ environment. Sample level ordination was calculated on rarefied (945 sequences, seed = 43980) genus-level aggregated data, which was then transformed with the centered log ratio method and reduced with principal components analysis for each intestinal part and layer to evaluate the beta diversity. Taxonomic distribution bar plot graphs and ordination graphs were created with the microViz (v0.9.0.9009)^60^⁠ and Matplotlib (3.5.2)^61^⁠⁠ packages, while alpha diversity box plot graphs were created with the ggplot2 (v3.3.6)^62^⁠⁠ package. Finally, we performed a differential abundance test with the ANCOM-BC (v1.4.0)^63^⁠ package, including independent variables in the formula and excluding features not observed in at least 10% of all samples. To see the full list of used R packages, consult Supplementary Table 2. Median log fold change values of differentially abundant taxa of the same genera were visualized as bar plots using python libraries Matplotlib (3.5.2) and pandas (1.4.3).^64^

Analysis of compositions of microbiomes with bias correction (ANCOM-BC) was performed using different levels of factors: metformin treatment status, sex, and diet type in each intestinal layer and part, *n* = 12 per group. To investigate the interaction between metformin treatment and the studied factors, analysis of compositions of microbiomes with bias correction (ANCOM-BC) was performed separately for each of the different combinations of factor levels, contrasting Met+ and Met- samples, *n* = 3 per group. Features representing the same genus were combined, and medians of the LogFC of abundances were plotted in each of the analyzed contrasts (Figures 7–10 and Supplementary Figures S1–S4). Individual dots were included in the plots to show the genera consisting of multiple features and the distribution of LogFC for each of the features.⁠

## Supplementary Material

Supplemental Material
