# Supplementary figures and images for: Spatial variation of the gut microbiome in response to long-term metformin treatment in high-fat diet-induced type 2 diabetes mouse model of both sexes
Source: Gut Microbes. 2023 Mar 16;15(1):2188663. doi: 10.1080/19490976.2023.2188663 (PMC10026874; doi:10.1080/19490976.2023.2188663)

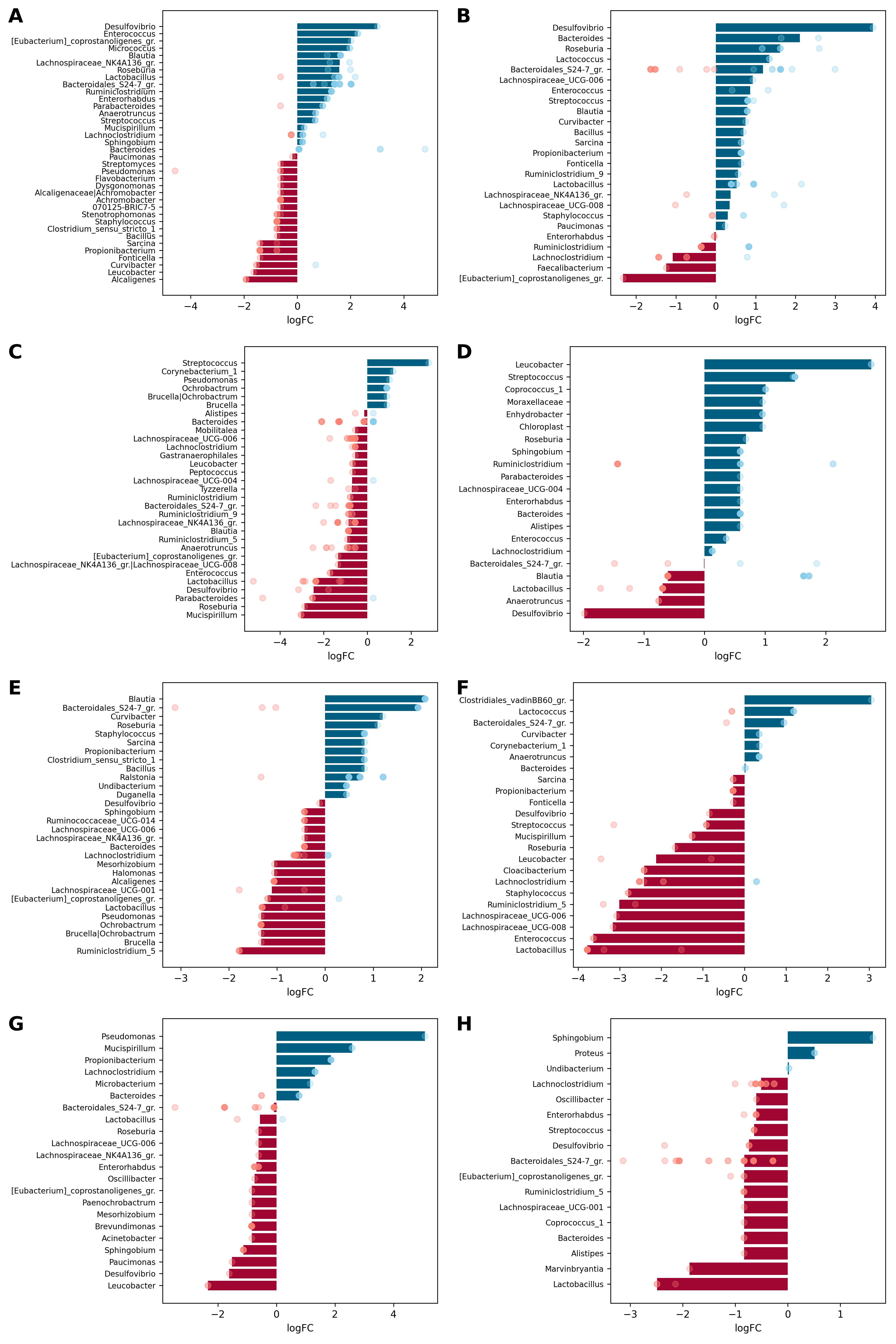

Supplement: Supplemental Material [file KGMI_A_2188663_SM9231.zip › Supplementary_materials/Supplementary_Figure_1.tiff]

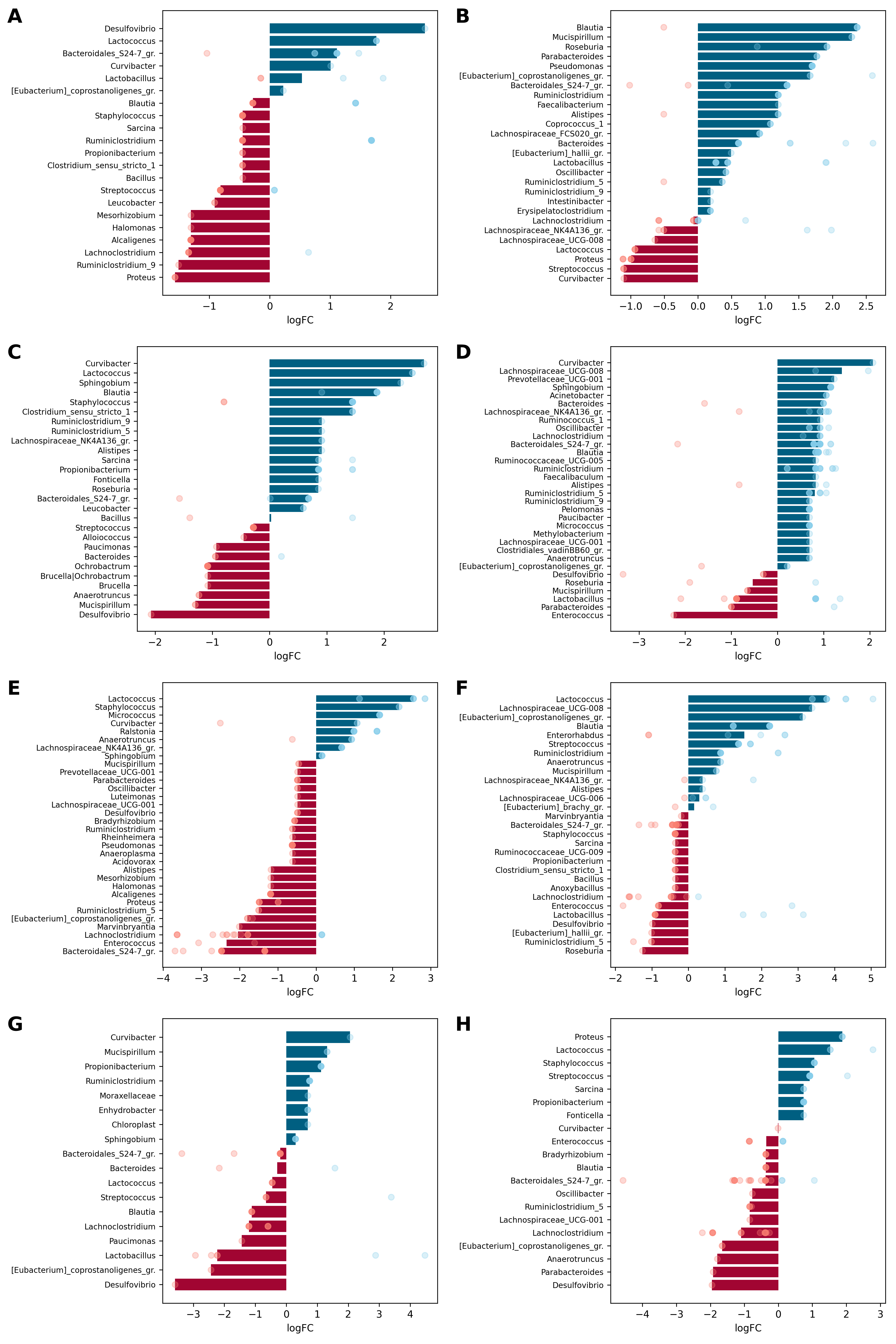

Supplement: Supplemental Material [file KGMI_A_2188663_SM9231.zip › Supplementary_materials/Supplementary_Figure_2.tiff]

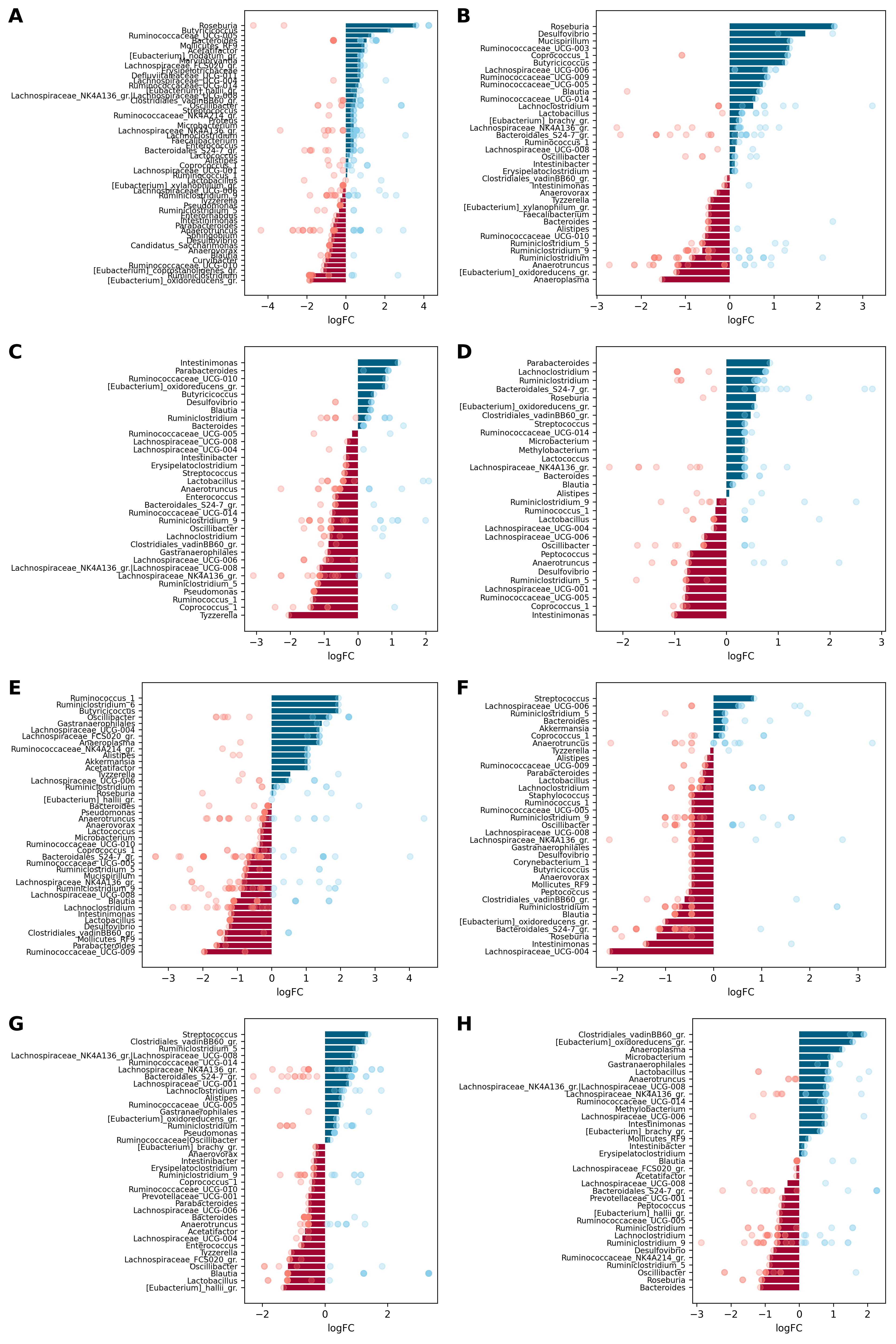

Supplement: Supplemental Material [file KGMI_A_2188663_SM9231.zip › Supplementary_materials/Supplementary_Figure_3.tiff]

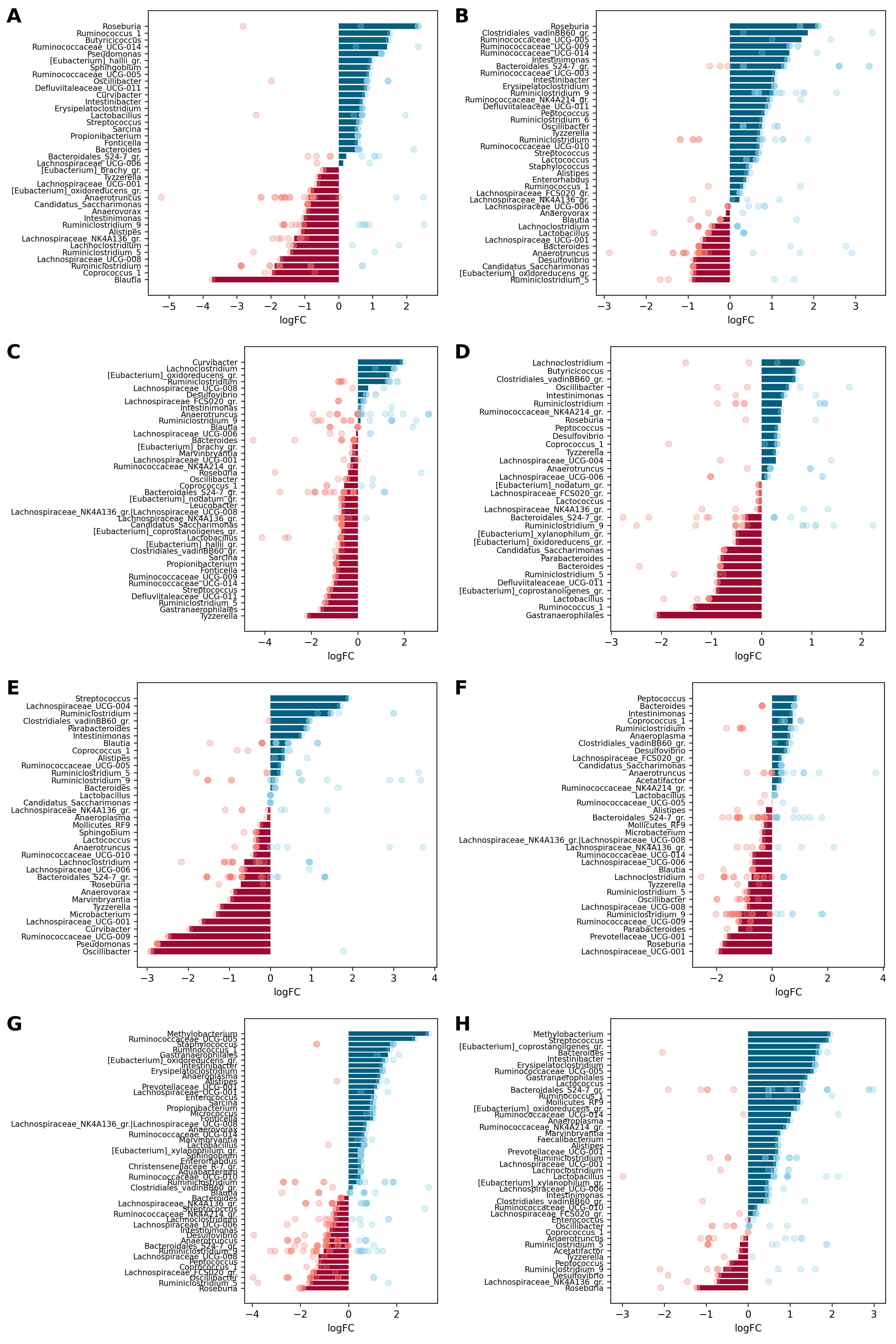

Supplement: Supplemental Material [file KGMI_A_2188663_SM9231.zip › Supplementary_materials/Supplementary_Figure_4.tiff]
