# Supplementary material for: Spatial variation of the gut microbiome in response to long-term metformin treatment in high-fat diet-induced type 2 diabetes mouse model of both sexes
Source: Gut Microbes. 2023 Mar 16;15(1):2188663. doi: 10.1080/19490976.2023.2188663 (PMC10026874; doi:10.1080/19490976.2023.2188663)
Supplement: Supplemental Material [file KGMI_A_2188663_SM9231.zip › Supplementary_materials/Supplementary_Figure_legends.doc]

**Supplementary Figures**

Supplementary Figure 1. Differentially abundant genera in response to metformin treatment in the proximal small intestine (expressed as LogFC): (A) mucosa of high-fat diet-fed male mice; (B) lumen of high-fat diet-fed male mice; (C) mucosa of high-fat diet-fed female mice; (D) lumen of high-fat diet-fed female mice; (E) mucosa of control diet-fed male mice; (F) lumen of control diet-fed male mice; (G) mucosa of control diet-fed female mice; (H) lumen of control diet-fed female mice. Blue bars represent genera with increased abundance among metformin users, and red bars – with decreased abundance. Dots of the corresponding color indicate all the individual features assigned to the genus.

Supplementary Figure 2. Differentially abundant genera in response to metformin treatment in the distal small intestine (expressed as LogFC): (A) mucosa of high-fat diet-fed male mice; (B) lumen of high-fat diet-fed male mice; (C) mucosa of high-fat diet-fed female mice; (D) lumen of high-fat diet-fed female mice; (E) mucosa of control diet-fed male mice; (F) lumen of control diet-fed male mice; (G) mucosa of control diet-fed female mice; (H) lumen of control diet-fed female mice. Blue bars represent genera with increased abundance among metformin users, and red bars – with decreased abundance. Dots of the corresponding color indicate all the individual features assigned to the genus.

Supplementary Figure 3. Differentially abundant genera in response to metformin treatment in the cecum (expressed as LogFC): (A) mucosa of high-fat diet-fed male mice; (B) lumen of high-fat diet-fed male mice; (C) mucosa of high-fat diet-fed female mice; (D) lumen of high-fat diet-fed female mice; (E) mucosa of control diet-fed male mice; (F) lumen of control diet-fed male mice; (G) mucosa of control diet-fed female mice; (H) lumen of control diet-fed female mice. Blue bars represent genera with increased abundance among metformin users, and red bars – with decreased abundance. Dots of the corresponding color indicate all the individual features assigned to the genus.

Supplementary Figure 4. Differentially abundant genera in response to metformin treatment in the colon (expressed as LogFC): (A) mucosa of high-fat diet-fed male mice; (B) lumen of high-fat diet-fed male mice; (C) mucosa of high-fat diet-fed female mice; (D) lumen of high-fat diet-fed female mice; (E) mucosa of control diet-fed male mice; (F) lumen of control diet-fed male mice; (G) mucosa of control diet-fed female mice; (H) lumen of control diet-fed female mice. Blue bars represent genera with increased abundance among metformin users, and red bars – with decreased abundance. Dots of the corresponding color indicate all the individual features assigned to the genus.
